# A Diagnostic Dilemma of Dysphonia: A Case Report on Laryngeal Myasthenia Gravis

**DOI:** 10.7759/cureus.16878

**Published:** 2021-08-04

**Authors:** Asad Ali Khan, Muhammad Waleed Khan, Tehreem A Kundi, Abdul Wali Khan, Zeeshan M Ali-Qazalbash

**Affiliations:** 1 Internal Medicine, Khyber Teaching Hospital, Peshawar, PAK; 2 Internal Medicine, College of Physicians and Surgeons Pakistan, Peshawar, PAK; 3 Internal Medicine, Hayatabad Medical Complex Peshawar, Peshawar, PAK

**Keywords:** myasthenia gravis (mg), laryngeal myasthenia gravis, late-onset myasthenia gravis, dysphonia, neuromuscular diseases

## Abstract

An autoimmune neuromuscular junction disorder, myasthenia gravis, occurs when antibodies are produced against postsynaptic membrane acetylcholine receptors. Late-onset myasthenia gravis, a rare variant of the disease found in the elderly, has become a diagnostic challenge on account of its atypical presentation. We proffer a case of a 60-year-old man that presented with progressive dysphonia and weakening of cough, which was eventually followed by difficulty in swallowing and nasal regurgitation. Examination and laboratory workup came out unremarkable apart from a positive acetylcholine receptor antibody test, due to which a diagnosis of laryngeal myasthenia, an uncommon presentation of late-onset myasthenia gravis was made. Following treatment with pyridostigmine and prednisolone saw a relief of the active complaints. This article highlights the need for physicians to stay alert and have a high suspicion of such probability for timely diagnosis.

## Introduction

Myasthenia gravis, an autoimmune neuromuscular junction disorder, occurs due to the production of antibodies against the acetylcholine receptors on the postsynaptic membranes [1]. About one-third of the cases present over the age of 50 years, and these constitute late-onset myasthenia gravis [2,3]. The disease presents with generalized or focal (ocular, bulbar) muscle weakness with fluctuation and fatigability as characteristic features. Additionally, voice changes can be the initial or, in rare instances, the only predominant complaint, such as in patients of laryngeal myasthenia gravis [4,5]. Such patients present with dysphonia and/or hoarseness that is commonly attributed to normal aging or other more common etiologies, leading to a missed or delayed diagnosis. This article delineates one such diagnostic challenge with an atypical presentation that was managed in a timely fashion and as a result, had a good clinical outcome.

## Case presentation

A 60-year-old man with a past medical history of hypertension and ischemic heart disease presented to the outpatient department of a tertiary care hospital in Peshawar with major complaints of progressive dysphonia and weakening of cough for the last one month.

The patient initially developed progressive and bothersome impairment in his speech, leading to dysphonia and slurring. This was followed by mild and intermittent difficulty in swallowing, along with nasal regurgitation and a sensation of food getting stuck in the throat, while there was no reported impairment in conscious level or fits.

Upon further evaluation, his vitals were stable, with a BP of 130/80 mmHg, a pulse of 84 beats/min, and a temperature of 98.6 F. A thorough neurologic examination was meticulously performed, that did not reveal any focal neurologic deficits. In addition, all the cranial nerves were intact, with a normal gag reflex and no diplopia or tongue fasciculations. However, his cough intensity declined with repeated coughing and his voice became hoarse. On examination of the limbs, bulk, tone, and power were all found to be normal while deep tendon reflexes were noted to be 2+ and both plantars were downgoing. All sensory modalities i.e., pain, touch, temperature, pressure, and proprioception were normal and the patient had a normal gait and cerebellar functions. Furthermore, examination of respiratory, cardiovascular, and gastrointestinal systems was unremarkable.

**Figure 1:**
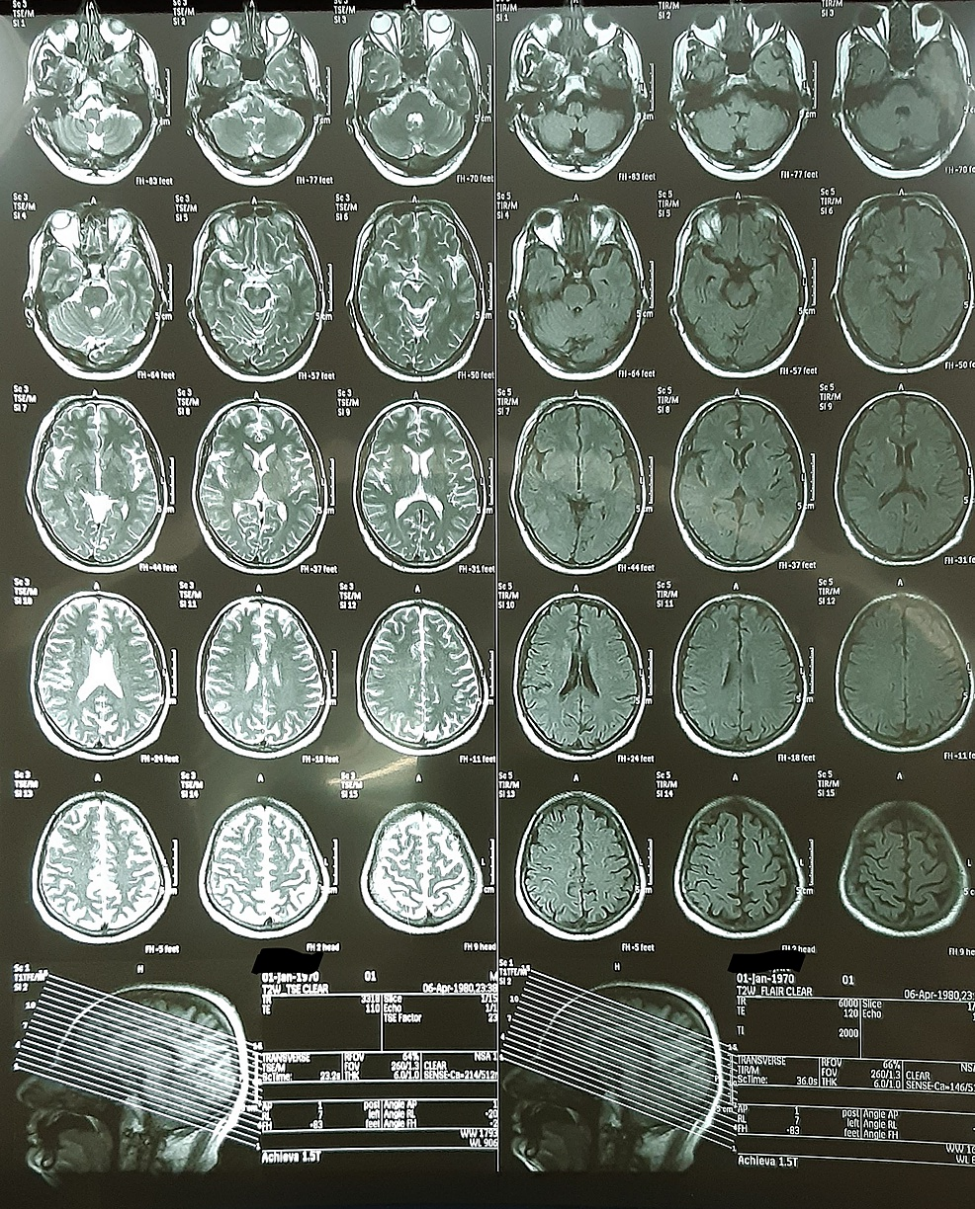
Showing transverse sections of a T2 weighted MRI- No abnormal findings present.

**Figure 2:**
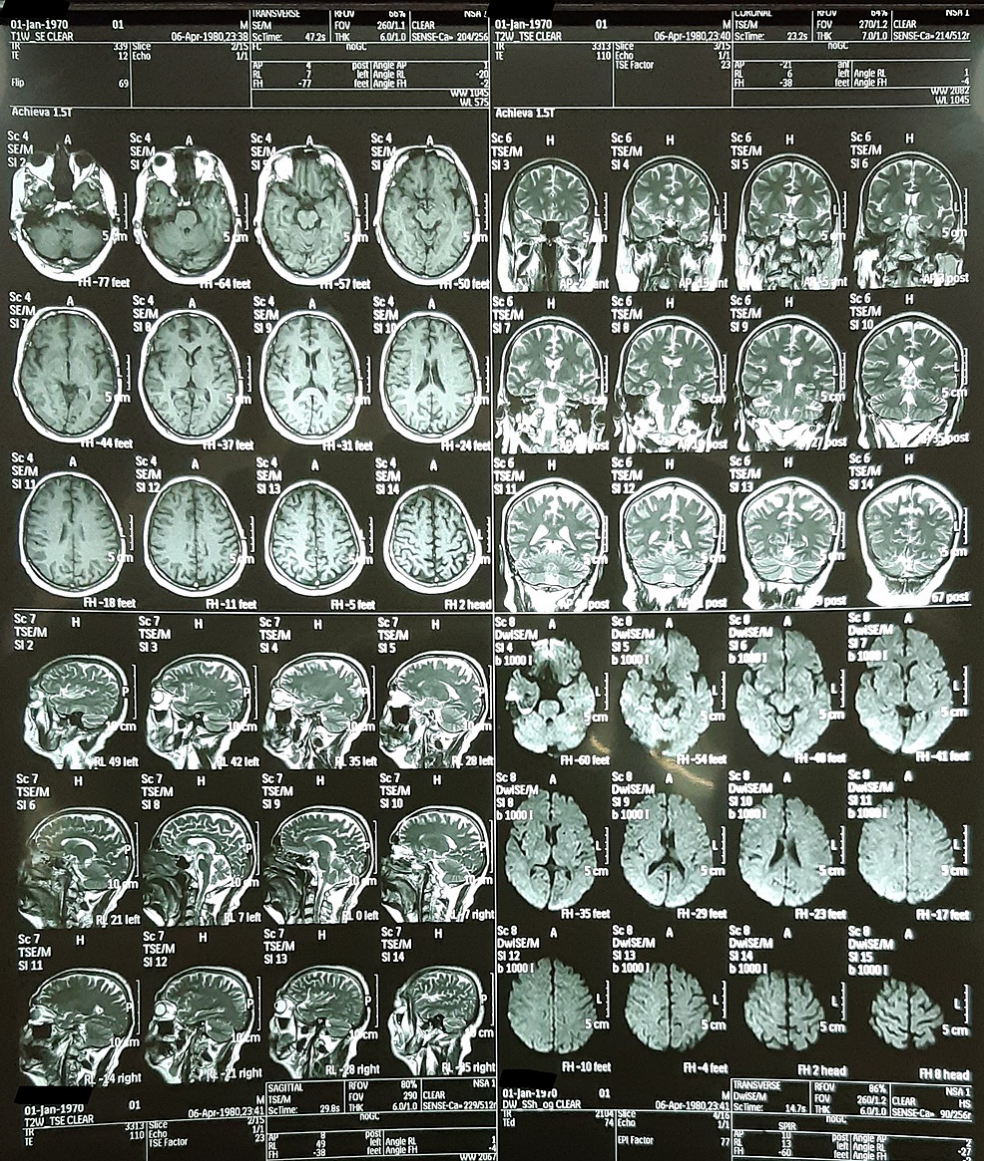
Showing sagittal sections of a T1 weighted MRI- No abnormal findings noted.

**Figure 3:**
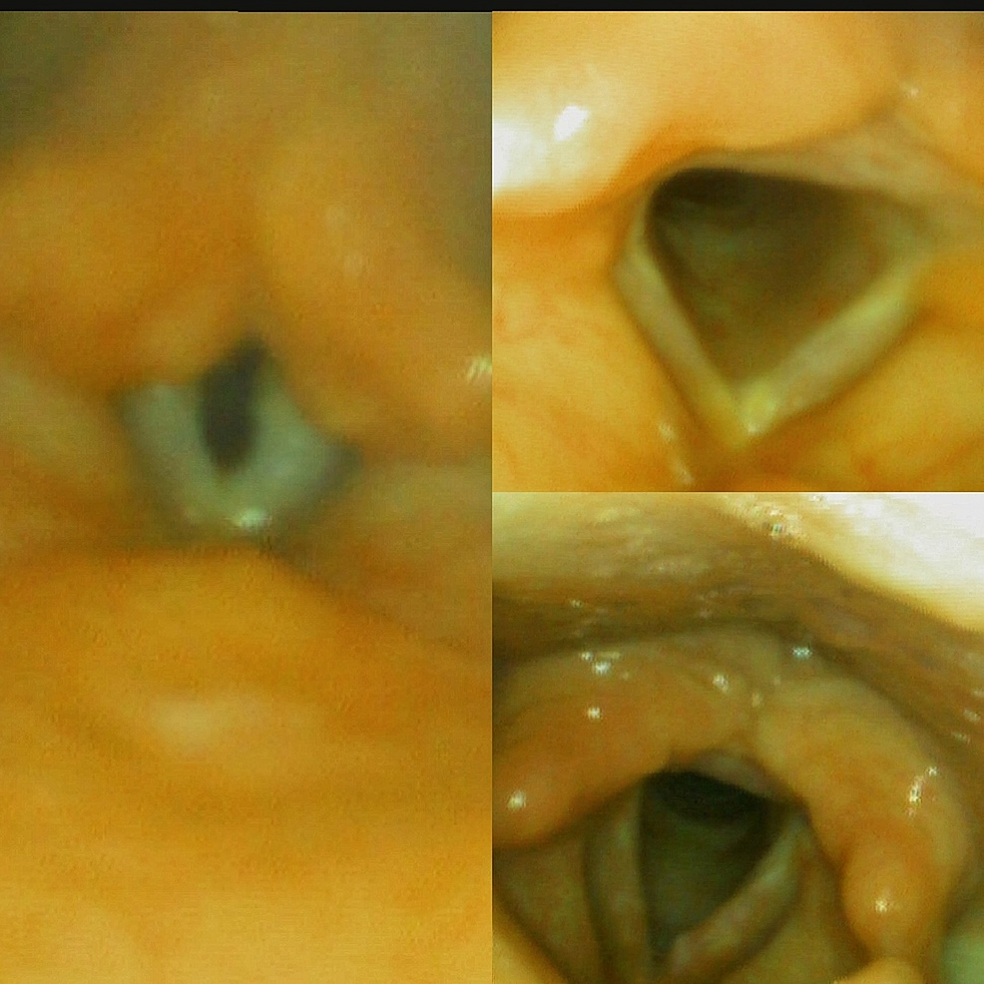
Laryngoscopy with normal findings.

Keeping the old age of the patient and his risk factors in mind, an urgent CT of the brain was ordered which ruled out intracranial hemorrhage. Additionally, all the subsequent investigations including cerebrospinal fluid (CSF) analysis for the pharyngeal-cervical-brachial variant of Guillain-Barré syndrome (GBS), nerve conduction studies (Table 1), MRI of the brain to exclude ischemic stroke and other central nervous systems (CNS) etiologies as shown in Figures 1, 2], and video laryngoscopy (Figure 3) returned normal.

**Table 1:** Nerve conduction study.

Nerve tested	Time of Recording	1^st^ stimulus potential (mv)	4^th^ stimulus potential		10^th^ stimulus potential	
Radial Nerve	At rest		mv	Decrease	mv	Decrease
		7.14	6.52	9%	6.7	6%
	Immediately after activation	10.49	9.67	8%	9.71	7%
	1 minute after activation	7.14	6.61	7%	6.87	4%
	3 minutes after activation	7.56	6.88	9%	7.14	6%
Facial Nerve	At rest	0.3	0.49	-63%		
	Immediately after activation	0.23	0.44	-91%		
	1 minute after activation	0.32	0.46	-44%		
	3 minutes after activation	0.4	0.56	-40%		
Conclusion: This Nerve conduction study reports normal findings and lacks the typical decremental responses seen in Myasthenia gravis						

A comprehensive list of routine investigations was advised, the overview of which is as follows: (Table 2).

**Table 2:** Laboratory investigations.

Parameter	Result	Reference Range
Hemoglobin	15.2 g/dL	11.5 – 17.5 g/dL
Total leukocyte count	10.2×10^3^/µL	4 – 11×10^3^/µL
Platelets	221×10^3^/µL	150 – 450×10^3^/µL
Sodium	137 mmol/L	135 – 150 mmol/L
Potassium	4.39 mmol/L	3.5 – 5.1 mmol/L
Chloride	109 mmol/l	96 – 112 mmol/L
Blood urea nitrogen	48.6 mg/dL	10 – 50 mg/dL
Serum creatinine	0.9 mg/dL	0.64 – 1.2 mg/dL
Random blood sugar	112 mg/dL	70 – 140 mg/dL
HbsAg and Anti-HCV	Negative	Cutoff rate: 1.00 (HCV and HBsAg) Patient rate: 0.04 (HCV) 0.77 (HbsAg)
Alkaline phosphatase	89 U/L	40 – 129 U/L
Alanine transaminase	23 U/L	10 – 50 U/L
Triglycerides	111 mg/dL	<200 mg/dL
Total cholesterol	215 mg/dL	<200 mg/dL
LDL cholesterol	144 mg/dL	<150 mg/dL
HDL cholesterol	45 mg/dL	35 – 65 mg/dL

A possibility of myasthenia gravis was considered and an acetylcholine receptor antibody test was ordered, which came back positive i.e., 69.4 nmol/L (>0.5 nmol/L is positive). Thus, a diagnosis of laryngeal Myasthenia Gravis- a presentation of late-onset bulbar Myasthenia Gravis- was made and the patient was started on Tablet Pyridostigmine 60 mg, two tablets four times a day with oral prednisolone 20 mg/day which was tapered off later on. At his first monthly follow-up, the patient showed significant improvement, with relief of dysphagia and improvement in speech. Following which a CT of the thorax was done but the report found no thymic pathology.

## Discussion

Myasthenia Gravis is an autoimmune disorder that occurs due to IgG autoantibodies against the postsynaptic acetylcholine receptors on skeletal muscles resulting in impaired transmission at the neuromuscular junctions [1].

Although the distribution of the diseases varies widely, the worldwide estimated incidence ranges from 0.3 to 2.8 per 100,000 and the median prevalence is 10 per 100,000 [6].

Although most cases of Myasthenia Gravis occur in relatively younger individuals, those that occur after the age of 50 years constitute late-onset myasthenia gravis (LOMG), accounting for about one-third of the total patients of Myasthenia gravis [2,3]. Only a small group of such patients presents with isolated bulbar symptoms including chewing & swallowing difficulties and voice changes [7-9].

Laryngeal myasthenia gravis is a very rare variant of late-onset myasthenia gravis with only a few cases described so far and thus requires special consideration [4,5]. Patients initially present with voice changes such as hoarseness, dysphonia, the nasal twang in voice, etc. due to weakness of palatal and laryngeal muscles. This poses obvious diagnostic challenges as the symptoms can be attributed to normal changes of aging or other more common etiologies including neurologic (Stroke, Parkinson's disease, GBS, etc.) & vocal cord (benign & malignant lesions) disorders. This form of the disease may lack the fatigability seen in the classic Myasthenia Gravis. Our patient was one of such cases who presented with dysphonia.

The overlapping and atypical nature of symptoms often leads to underdiagnosis or misdiagnosis in old patients [10]. Patients with clinical suspicion need prompt evaluation to avoid delay in the diagnosis and to prevent complications such as myasthenic crisis or acute respiratory failure. The clinical features, symptoms improvement with edrophonium administration, and normal laryngeal examination guide further workup. Electrodiagnostic tests such as repetitive nerve stimulation and single-fiber electromyography should be performed if available. Anti-AchR antibodies are highly specific for Myasthenia gravis but can be absent in some patients especially those with localized myasthenia gravis, mild disease, or remission [11]. In such patients, muscle tyrosine kinase antibodies (MuSK) should be ordered.

The patient under consideration was a difficult case to diagnose, especially given his old age. He had a normal otorhinolaryngological evaluation (Figure 3) and no neurologic cause of his illness.

A normal nerve conduction study further distracted the case. Still, the anti-Ach-R antibody test was ordered that led to the diagnosis.

## Conclusions

Change in one’s voice is a possible presentation of myasthenia gravis, especially in older patients as is espoused by previous literature, but the rarity of the condition poses a significant diagnostic challenge, one which is further complicated by the atypical presentation of this condition as well as the possibility of other causes being responsible for this finding. This case alerts the physicians to be cognizant of and have a high suspicion for such probability.

## References

[1] Drachman DB (1994). Myasthenia gravis. N Engl J Med.

[2] Alkhawajah NM, Oger J (2013). Late-onset myasthenia gravis: a review when incidence in older adults keeps increasing. Muscle Nerve.

[3] Aragonès JM, Bolíbar I, Bonfill X, Bufill E, Mummany A, Alonso F, Illa I (2003). Myasthenia gravis: a higher than expected incidence in the elderly. Neurology.

[4] Mao VH, Abaza M, Spiegel JR, Mandel S, Hawkshaw M, Heuer RJ, Sataloff RT (2001). Laryngeal myasthenia gravis: report of 40 cases. J Voice.

[5] Michalska T, Rowińska-Marcińska K, Strugalska H, Maniecka-Aleksandrowicz B, Emeryk-Szajewska B (1996). Myasthenia preceded by dysphonia. Clinical and electrophysical study (Article in Polish). Neurol Neurochir Pol.

[6] Deenen JC, Horlings CG, Verschuuren JJ, Verbeek AL, van Engelen BG (2015). The epidemiology of neuromuscular disorders: a comprehensive overview of the literature. J Neuromuscul Dis.

[7] Sharp HR, Degrip A, Mitchell DB, Heller A (2001). Bulbar presentations of myasthenia gravis in the elderly patient. J Laryngol Otol.

[8] Basiri K, Ansari B, Okhovat AA (2015). Life-threatening misdiagnosis of bulbar onset myasthenia gravis as a motor neuron disease: how much can one rely on exaggerated deep tendon reflexes. Adv Biomed Res.

[9] Yang X, Niu L, Yang C, Wang L, Liu J, He G (2019). Clinical features of laryngeal myasthenia gravis: a case series. Am J Otolaryngol.

[10] Vincent A, Clover L, Buckley C, Grimley Evans J, Rothwell PM (2003). Evidence of underdiagnosis of myasthenia gravis in older people. J Neurol Neurosurg Psychiatry.

[11] Pasnoor M, Dimachkie MM, Farmakidis C, Barohn RJ (2018). Diagnosis of myasthenia gravis. Neurol Clin.

